# K Pump-Mackenzie: Technological Development and Initial Bench Validation of a Fully Implantable 5G-Enabled Ventricular Assist Device

**DOI:** 10.21470/1678-9741-2026-0150

**Published:** 2026-06-12

**Authors:** Luiz Fernando Kubrusly, Osvaldo Malafaia, Benedito Guimarães Aguiar Neto, Paulo Batista Lopes, Roberto Coutinho, Cristiano Akamine, Pollyana Coelho da Silva Notargiacomo, Giovanna Santana, Cleide Lustosa de Oliveira da Silva, Elesandro Antonio Baptista, Licia M. D’arezzo Maestrelli, Douglas Mesadri Gewehr, Cris Rangel de Abreu, Fernando Bermudez Kubrusly

**Affiliations:** 1 Programa de Pós-Graduação em Princípios da Cirurgia, Faculdade Evangélica Mackenzie do Paraná, Curitiba, Paraná, Brazil; 2 Instituto Presbiteriano Mackenzie, São Paulo, São Paulo, Brazil

**Keywords:** Heart-Assist Devices, Assisted Circulation, Artificial Heart, Implantable Medical Device, Medical Telemetry.

## Abstract

**Introduction:**

Advanced heart failure is an important cause of mortality in Brazil. Limited access to heart transplantation and the high cost of imported devices reinforce the need for national technological solutions. The K Pump-Mackenzie project proposes the development of a fully implantable mechanical circulatory assist device, integrated with 5G telemetry, with potential application in the unified health system of Brazil, the Sistema Único de Saúde.

**Methods:**

A centrifugal prototype rotor, with real-time control system and integrated flow and pressure sensors, was developed. The device was submitted to laboratory tests to evaluate mechanical performance, operational stability, structural integrity, and connectivity via 5G network. The preclinical protocol in the sheep model was designed and approved by the Comissão de Ética no Uso de Animais, from the Faculdade Evangélica Mackezie do Paraná, in accordance with Conselho Nacional de Segurança Alimentar e Nutricional guidelines.

**Results:**

In bench tests, the device showed controlled rotation of up to 10,000 rpm, adjustable flow between 2 and 6 L/min, and average latency of < 20 ms in data transmission. Continuous operational stability was observed for up to 72 hours, with adequate functioning of the monitoring and safety systems.

**Conclusion:**

K Pump-Mackenzie demonstrated technical feasibility and satisfactory performance in initial bench validation. The planned preclinical stage will be fundamental to evaluate safety and biological performance, constituting a necessary step for future clinical application, and will take place in 2026.

## INTRODUCTION

**Table:** 

Abbreviations, Acronyms & Symbols	
ANVISA	= Agência Nacional de Vigilância Sanitária
CEUA	= Comissão de Ética no Uso de Animais
FEMPAR	= Faculdade Evangélica Mackenzie do Paraná
HF	= Heart failure
IPM	= Instituto Presbiteriano Mackenzie
ISO	= International Organization for Standardization
MCS	= Mechanical circulatory support
SUS	= Sistema Único de Saúde

Heart failure (HF) is a progressive and debilitating clinical condition, characterized by the inability of the heart to maintain a cardiac output adequate to the metabolic needs of the body. In Brazil, it is estimated that > 6 million people live with the disease, which is one of the main causes of hospitalization in adults over 60 years of age, with high rates of readmission and in-hospital mortality^[^1^,^2^]^. Despite pharmacological advances, many patients progress to advanced stages, in which therapeutic options are limited^[^3^]^.

Heart transplantation remains the standard treatment for patients with refractory end-stage HF. However, it is restricted by the scarcity of donors, prolonged waiting time, and clinical contraindications, and is performed in < 500 patients per year in Brazil^[^4^]^. From this perspective, mechanical circulatory support (MCS) stands out as a viable alternative, either as a bridge to transplantation, for myocardial recovery, or as a destination therapy^[^5^.^6^]^.

However, the commercially available models are imported, with high cost and limitations regarding portability, energy autonomy, and integration with remote monitoring systems^[^7^]^. These barriers hinder its widespread adoption in the unified health system (or Sistema Único de Saúde [SUS]) of Brazil and accentuate inequalities in access to advanced therapies.

In order to fill this gap, in June 2000, the first model of the K Pump was developed, at that time with axial flow characteristics, in small size (30 cc and 7 cm long). Despite these characteristics, it was able to generate flows between 5 and 8 L/min, operating without causing hemolysis in bovine blood, reason for our publication in the Brazilian Journal of Cardiovascular Surgery^[^8^]^. After this initial experience, the axial model was abandoned due to the high cost in developing and balancing the rotor. We then began developing the K Pump Fecial centrifugal pump model, which offered acceptable flow rates with low fuel consumption (2009). From this model the pump was submitted to a variety of modifications mainly in the rotor, based on computational fluid dynamics with the result of an output of 4,95 L/min and efficiency around 10% with 1,79 w (K Pump Mehta).

In 2023, through a substantial research funding of the Financiadora de Estudos e Projetos (or Finep) obtained by the Instituto Presbiteriano Mackenzie (IPM), the project took a promising course. The K Pump-Mackenzie device, a fully implantable MCS, with centrifugal architecture and remote control based on 5G technology, began its development. The device is the result of a collaboration between Brazilian physicians, engineers, and researchers from IPM and proposes itself as an innovative, accessible solution with potential application in the context of public health^[^9^]^. This article presents the technological development and results of the initial bench validation of the K Pump-Mackenzie, in addition to the description of the preclinical protocol planned in an animal model, a necessary step for future clinical application in the treatment of advanced HF in Brazil.

## METHODS

### Study Design

This is a translational experimental study divided into two phases: (1) technological development and benchtop validation of the fully implantable centrifugal mechanical circulatory assist device (K Pump-Mackenzie); and (2) preclinical validation in an animal model

### Phase 1 - Technological Development of the Device

#### Device Design and Architecture

The K Pump-Mackenzie was designed as a continuous-flow centrifugal pump intended for left ventricular support. The system consists of a pump body, a rotor with brushless direct current motor, integrated flow, differential pressure, and temperature sensors, a telemetry unit, and an electronic control module (Figures 1 and 2).

**Fig. 1:**
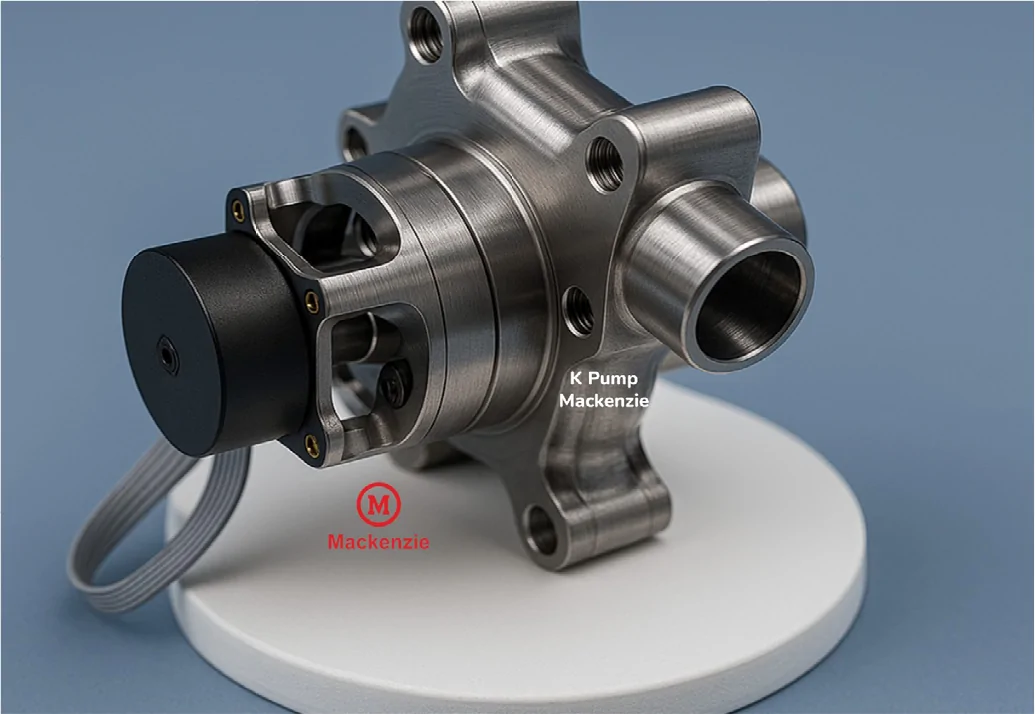
K Pump-Mackenzie. Source: Cristiano Akamine, 2025.

**Fig. 2:**
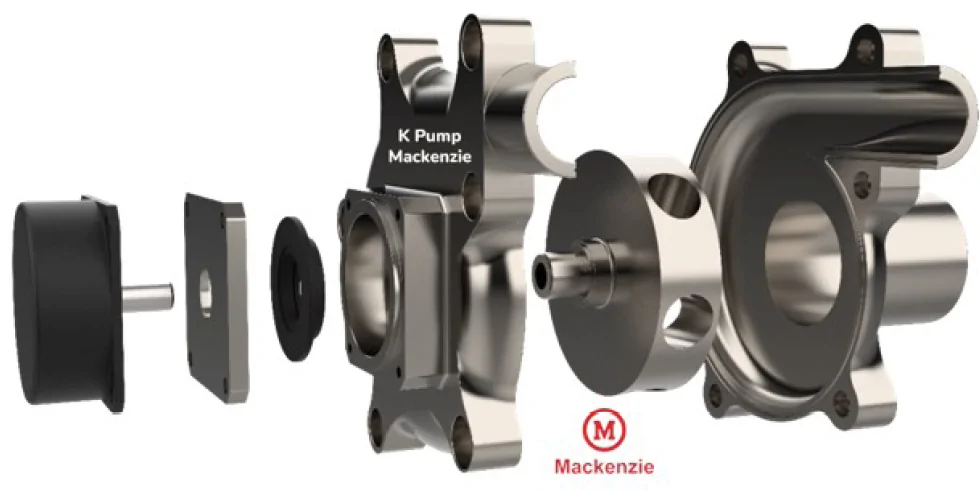
K Pump-Mackenzie - exploded view.

The architecture was developed with a focus on miniaturization, with 4.5 cm at its widest diameter, hydrodynamic efficiency, and total integration for intrathoracic implants. The modulation of rotational velocity allows adjustment of the output according to simulated hemodynamic demand.

#### Materials and Characterization

The mechanical structure was predominantly made of Ti6Al4V ELI titanium alloy, medical grade, according to Advancing Standards Transforming Markets F136 and International Organization for Standardization (ISO) 5832-3 specifications. The selection of the material was based on properties of mechanical strength, low density, and corrosion resistance.

The elemental characterization of the alloy was performed by energy dispersive X-ray spectroscopy. Biocompatibility was planned according to ISO 10993 guidelines, including cytotoxicity, sensitization, and systemic toxicity assays.

As a strategy for superficial modification, plasma thermal spraying was used in the areas of greater hematic contact, aiming to improve hemocompatibility.

#### Electronic System and Telemetry

The electronic system was developed to operate with low-latency 5G connectivity, allowing real-time remote monitoring and control. The graphical interface is designed for continuous visualization of operating parameters.

Tests of connectivity, network stability, structural integrity, and fault simulation were carried out in a controlled environment.

#### Bench Tests

Laboratory tests included:

a) Rotational stability evaluation
b) Flow measurement under different simulated resistance conditions
c) Structural integrity testing
d) Electromagnetic compatibility testing
e) Simulating firmware failures

The assays were conducted in a controlled environment with blood fluid simulation.

### Phase 2 - Preclinical Validation in Animal Model

The preclinical phase consists of an experimental study in a sheep model (*Ovis aries*), with the objective of evaluating safety and functional performance of the implanted device. The protocol was approved by the Comissão de Ética no Uso de Animais (CEUA) from Faculdade Evangélica Mackenzie do Paraná (FEMPAR) in accordance with the guidelines of Conselho Nacional de Segurança Alimentar e Nutricional (Law No. 11,794/2008).

Two clinically healthy animals, weighing between 60 and 70 kg, will be submitted to general anesthesia and lateral thoracotomy and partial extracorporeal circulation for implantation of the device with partial cardiac output support.

The pump will be implanted through the apex of the left ventricle, remaining in the ventricular cavity, with the rotor supported by the diaphragm and connected to the ascending aorta via a Dacron® tube through direct anastomosis, similar to pumps already available such as HeartMate™ III and HeartWare™. Intraoperative monitoring will include invasive blood pressure, heart rate, peripheral saturation, and cardiac output (Swan-Ganz).

In the postoperative period, the animals will be followed for up to 72 hours, with continuous clinical evaluation, serial laboratory tests (blood count, kidney and liver function, inflammatory markers), cardiac output, and also continuous remote monitoring via 5G network. The main objective of this experiment will be to evaluate hemolysis and the animal's ability to maintain adequate cardiac output. The decrease in cardiac output will be achieved by administering high doses of beta-blockers (proof-of-concept).

At the end of the follow-up, the animals will be euthanized. Samples of the target organs (heart, lungs, kidneys, liver, spleen) and tissues adjacent to the implant will be processed for histopathological and immunohistochemical analysis, with evaluation of necrosis, fibrosis, thrombogenicity, and inflammatory response.

### Statistical Analysis

Quantitative data are expressed as mean ± standard deviation or median and interquartile range, according to distribution. Appropriate parametric or non-parametric tests are used, with a significance level of *P* < 0.05. The analyses are performed using R or SPSS software.

## RESULTS

### Phase 1 - Bench Validation

The prototype showed operational stability in a controlled test environment (bench test). The maximum speed reached 10,000 rpm, with an adjustable flow range between 2 and 6 L/min under simulated systemic resistance conditions. The electronic system demonstrated average latency of < 20 ms in the 5G connectivity tests. Data transmission occurred continuously, with stable remote monitoring of operational parameters.

Structural integrity tests showed no deformities or mechanical failures under continuous operation. The device maintained uninterrupted operation for up to 72 hours in continuous test cycles. Component failure simulations have properly enabled the security mechanisms programmed into the firmware. The electromagnetic compatibility tests demonstrated system stability in a controlled environment. These results allow the safe transition to the experimental phase in an animal model.

### Phase 2 - Preclinical Validation

The preclinical phase will be in progress at the time of submission of this manuscript. The experimental protocol was initiated according to the schedule approved by CEUA (FEMPAR). The data regarding the implantation and longitudinal follow-up of the animals will be presented in a subsequent publication, after completion of the follow-up period and complete histopathological analysis. K Pump-Mackenzie thoracic implants will be performed in adult sheep, with clinical, laboratory, and functional monitoring for up to 48 - 72 hours.

The device is expected to feature:

• Continuous functionality with maintenance of effective cardiac output;
• Hemodynamic stability of the animals without the need for additional support;
• Anatomical integration without compressions or displacements;
• Absence of signs of systemic infection, organ failure, or severe bleeding;
• Stable real-time data transmission via 5G network, with automatic alerts;
• Histopathological results compatible with satisfactory biocompatibility (absence of significant thrombi, necrosis, or extensive fibrosis in the tissues adjacent to the implant).

The systematic collection of clinical and histological data will allow the evaluation of the safety, inflammatory response, and functional performance of the device, supporting the regulatory registration stage and, subsequently, submission to the Agência Nacional de Vigilância Sanitária (ANVISA).

## DISCUSSION

The development of K Pump-Mackenzie represents a strategic advance in the search for affordable and effective national solutions for the treatment of end-stage HF in Brazil specifically for our unified health system (the SUS). In a scenario marked by the scarcity of organs for transplantation and the high cost of imported devices, the creation of a fully implantable MCS, with remote control by 5G technology, is configured as an innovation with high potential for clinical and economic impact.

The functional characteristics obtained in the bench phase, such as rotation control, real-time response, and flow stability, are compatible with internationally renowned devices, such as the HeartMate™ III, which has shown survival rates of > 80% in two years of use^[^6^]^ (despite our model K Pump-Mackenzie does not use magnetic levitation), and, in addition, the use of 5G technology integrated into the device introduces an unprecedented layer of connectivity in the national scenario, allowing continuous remote monitoring, personalization of circulatory support, and the possibility of immediate response to adverse events^[^10^]^.

The experimental model in sheep, adopted in this project, follows international references and is widely validated in the literature as an adequate translational model for implantable cardiovascular devices^[^11^]^. The planned evaluation for up to 72 hours, followed by a new experiment of 90 days, will allow not only to measure functional and clinical parameters, but also to understand the biological effects of the device in direct contact with living tissues, which is essential for the regulatory stage with ANVISA.

Although the *in vitro* and simulated results are promising, it is recognized that the preclinical phase is limited by the restricted number of animals and the short initial observation period. Such limitations will be addressed in future studies, with larger samples and prolonged follow-up. In addition, aspects such as driveline infection, thoracic adaptation, and local immune response should be carefully analyzed, as already observed in studies with international devices^[^5^.^7^]^.

From the point of view of application in the SUS, the initial costs of development and implementation represent an important issue. However, national production, local maintenance, and the reduction of prolonged hospitalizations can generate savings in the medium term, as already suggested by economic models applied to similar devices in the United States of America and Europe^[^7^.^9^]^.

Thus, K Pump-Mackenzie emerges as a viable, innovative, and potentially disruptive alternative, in line with the Brazilian reality and with global guidelines for expanding access to cardiovascular health technology. The continuity of experimental validation and the strengthening of articulation with regulatory agencies will be decisive for the transition of the device to clinical practice.

## CONCLUSION

K Pump-Mackenzie is a centrifugal flow MCS, fully deployable and integrated with 5G telemetry, developed with national technology. The bench tests demonstrated technical feasibility, operational stability, and adequate performance of the mechanical and electronic systems. The preclinical validation in a sheep model is in progress and will be decisive to confirm safety and functional performance, a necessary step for future clinical applications.

## Data Availability

The authors declare that the data supporting the findings of this study are available within the article.

## Preprint

https://doi.org/10.1590/SciELOPreprints.15302
